# HLA expression in human hepatocellular carcinoma.

**DOI:** 10.1038/bjc.1988.84

**Published:** 1988-04

**Authors:** A. C. Paterson, R. Sciot, M. C. Kew, F. Callea, G. M. Dusheiko, V. J. Desmet

**Affiliations:** Experimental Pathology Unit, School of Pathology, South African Institute for Medical Research, Johannesburg.

## Abstract

**Images:**


Br. J. Cancer (1988), 57, 369–373 © The Macmillan Press Ltd., 1988

### HLA expression in human hepatocellular carcinoma

A.C. Paterson[1], R. Sciot[2], M.C. Kew[3], F. Callea[4], G.M. Dusheiko[3] & V.J. Desmet[2]

[1]*Experimental Pathology Unit, School of Pathology, South African Institute for Medical Research and University of the Witwatersrand, PO Box 1038, Johannesburg 2000, South Africa;* [2]*Laboratory of Histo- and Cytochemistry, Department of Pathology, U.Z. St Rafaël, Catholic University of Leuven, B-3000 Leuven, Belgium;* [3]*Department of Medicine, University of the Witwatersrand Medical School, York Road, Parktown 2193, Johannesburg; and* [4]*Histochemistry Unit, First Department of Pathology, Spedali Civili of Brescia, 25100 Brescia, Italy.*

**Summary** This study examines the expression of MHC class I and II antigens and their related invariant chains in 70 cases of human hepatocellular carcinoma (HCC), using monoclonal (Mabs) and polyclonal antibodies. In comparison with normal hepatocytes, the majority (94.3%) of HCCs show enhancement or acquisition of HLA-A, B, C in either a cytoplasmic or membranous distribution, with staining being uniformly distributed throughout the specimen. HLA-A, B, C was accompanied by $\beta_2$-microglobulin expression in all but two cases. Although 44.9% of specimens showed HLA-DR expression, positively staining tumour cells were often sparse and heterogeneously distributed. By contrast, the invariant (I) chain, present in 47.1% of cases, was frequently intensively stained and extensive in distribution. HLA-DR staining was usually cytoplasmic although two cases showed faint membranous enhancement. In addition to HLA-DR and I-chain, two cases also showed HLA-DQ staining. Display of MHC antigens was not related to tumour differentiation or size of the lesion (resected *vs.* advanced tumours). It is possible that the acquisition of class I antigens by the majority of HCCs may influence tumour behaviour.

Further to their role in the restriction of the immune response in viral infection and transplantation, there is evidence to suggest that the products of the major histocompatibility complex (MHC) genes may influence tumour growth and metastasis. This relationship has been extensively studied *in vitro* and *in vivo* using animal models (Goodenow *et al.*, 1985), and may also contribute to the behaviour of certain human tumours (Fleming *et al.*, 1981; Ruiter *et al.*, 1986; Doyle *et al.*, 1985; Whelan *et al.*, 1985; van den Ingh *et al.*, 1987). The mechanism(s) by which the MHC antigens modify tumour behaviour still require clarification (Demant, 1986; Festenstein, 1987).

The class I antigens (HLA-A, -B, -C) are 45 kD transmembrane glycoproteins noncovalently associated with a 12 kD invariant light chain, $\beta_2$-microglobulin ($\beta_2$m) (Strachan, 1987). Their distribution is more restricted than was originally held (Daar *et al.*, 1984). The situation pertaining to hepatocytes is uncertain with some workers failing to detect them on normal liver cells (Ponder *et al.*, 1983; Lautenschlager *et al.*, 1984; Fukusato *et al.*, 1986) and others reporting their presence at low levels (Saunders *et al.*, 1979; Montano *et al.*, 1982; Nagafuchi *et al.*, 1985; Nagafuchi & Scheuer, 1986).

Class II antigens (HLA-DP, -DQ, -DR) comprise two noncovalently linked transmembrane polypeptides (Lafuse & David, 1984), associated for part of the cell cycle with another transmembranous glycoprotein, the invariant (I) chain (Jones *et al.*, 1978). Although their distribution is more widespread than originally recognised, it is generally agreed that hepatocytes do not express class II antigens in normal liver (Fukusato *et al.*, 1986). Both class I and class II products may be displayed by hepatocytes under a variety of pathological conditions, however (Nagafuchi *et al.*, 1985; Fukusato *et al.*, 1986; Nagafuchi & Scheuer, 1986; van den Oord *et al.*, 1986).

To date there is limited information available on HLA expression in hepatocellular carcinoma (HCC) with relatively few examples having been examined, either as solid tumours or cell lines (Natali 1981; Fukusato *et al.*, 1986; Mazzeo *et al.*, 1986). We have therefore studied the pattern of expression of HLA-A, -B, -C and $\beta_2$m, as well as HLA-DQ, HLA-DR and the associated I chain, using appropriate monoclonal and polyclonal antibodies.

Correspondence: A.C. Paterson.
Received 9 April 1987; and in revised form, 8 December 1987.

### Materials and methods

### *Human tissues*

The series comprised 70 samples of hepatocellular carcinoma from 56 black and 14 caucasian patients (66 male, 4 female) Hepatitis B viral (HBV) status and serum alpha-foetoprotein (AFP) levels were known in 60 cases.

Fresh tissue obtained at autopsy performed within 1 h of death (32 cases), by needle biopsy (26), or during resection of small tumours (12), was snap-frozen in OCT compound (Ames Co., Div. of Miles Laboratories, Elkhard) and stored at −70°C.

Histopathological diagnosis was made on routinely processed H&E sections. Tumours were graded as well, moderately or poorly differentiated according to the criteria of the World Health Organization (World Health Organization, 1978).

In view of the current uncertainty regarding the expression of class I antigens on normal hepatocytes, 10 snap-frozen samples of histologically normal liver were included as control material. These were obtained from patients under investigation for conditions other than HCC or HBV infection.

### *Monoclonal antibodies*

An anti-HLA class 1 Mab was obtained from Cappel Laboratories, Cochranville PA, (working dilution 1:50). This is a murine Mab of $IgG_1$ class directed against the 45,000 dalton polypeptide associated with $\beta_2$m, obtained by immunization of BALB/c mice with concavalin A-activated T-cell blasts followed by fusion of immune spleen cells with NS-1 myeloma cells (Cappel Product Information sheet). Mabs recognising nonpolymorphic regions of HLA-DR (anti-HLA-DR) and HLA-DQ (Leu-10), were obtained from Becton–Dickinson, Sunnyvale CA; (working dilutions 1:200 and 1:50 respectively). VIC-Y1 is a monoclonal antibody recognising the invariant (I) chain of HLA-DR (Quaranta *et al.*, 1984). Working dilution for VIC-Y1 was 1:200.

In addition, T-cell subsets were examined with a panel of commercially available Mabs: Leu-7 detecting killer and natural killer (NK) cells (Becton–Dickinson); OKT-4 reacting with helper/inducer T-cells (Ortho Pharmaceutical Co., Raritan, NJ); OKT-8 against suppressor/cytotoxic T-cells (Ortho). All were applied at a dilution of 1:10.

*Polyclonal antibodies*

Rabbit antibodies against the hepatitis B virus (HBV) surface antigen (anti-HBsAg), core antigen (anti-HBcAg), and human $\beta_2$m were purchased from Dako Immunoglobulins. They were applied for 30 min at dilutions of 1:50, 1:20 and 1:200 respectively.

*Immunoperoxidase techniques*

Cryostat sections (5 $\mu$m) were air-dried overnight at room temperature, fixed for 10 min in absolute acetone and stored wrapped in aluminium foil, at −20°C until use.

Monoclonal antibodies were demonstrated by a three step immunoperoxidase technique. Serial sections were rehydrated in PBS (pH 7.2) and incubated with the Mabs for 30 min at the dilutions specified above. The second and third steps comprised treatment for 30 min with rabbit-antimouse immunoglobulins (Igs) (Dako) at a dilution of 1:50, followed by peroxidase conjugated swine-antirabbit Igs (Dako), dilution 1:100, also for 30 min. Ten percent human AB serum was added to each, to reduce background staining. Each step was followed by vigorous washing in PBS. Sections were then incubated with 0.05% 3-amino-9-ethylcarbazole in 0.05 M acetate buffer (pH 4.9) and 0.01% $H_2O_2$. All staining was carried out at room temperature.

Polyclonal antibodies were used in a three-step unlabelled peroxidase-antiperoxidase procedure performed at room temperature. Sections were incubated with primary antibodies for 30 min at the dilutions specified above, This was followed by swine-anti rabbit Igs (dilution 1:100) and, thereafter, rabbit peroxidase-antiperoxidase complex (Dako; dilution 1:300), both for 30 min. Sections were washed in three changes of PBS between steps. The reaction product was developed with 3-amino-9-ethylcarbazole and $H_2O_2$. Negative controls consisted of omission of the primary antibody or use of the chromogen alone.

Slides were examined in batches according to primary antibody to allow later comparison between MHC products and their relevant invariant chains. Staining of HLA-A, -B, -C and $\beta_2$m was recorded as absent, cytoplasmic and membranous (±cytoplasmic). HLA-DR, -DQ and I chain staining were scored as 0 (absent), 1+ (<10% of hepatocytes positively staining), 2+ (10–50% positive), 3+ (>50% positive) and 4+ (>90% of cells positive).

### Results

Of 70 cases examined, 23 were well, 33 moderately and 14 poorly differentiated HCC. Twelve were resected specimens, staged as 'early' HCC, and the remainder represented advanced tumours. HBsAg and HBcAg were demonstrated by immunostaining in one case.

*Class I expression*

In addition to sinsoidal staining (endothelial and Kupffer cells), weak, membranous display of HLA-A, -B, -C was observed on variable numbers of hepatocytes in all 10 histologically normal livers (Figure 1).

Positive staining for HLA-A, -B, -C was present in 66 of 70 cases (94.3%). Of the 66 positive tissues, 41 displayed membranous staining accompanied by variable intracytoplasmic positivity (Figure 2), while 23 showed a granular intracytoplasmic stain, sometimes accompanied by sinusoidal enhancement (Figure 3). In 4 specimens the tumour was negative for the antigen although adjacent non-neoplastic hepatocytes showed enhanced membranous HLA-A, -B, -C display (Figure 4). Staining was of uniform consistency in positive specimens with the cytoplasmic or membranous pattern being conserved throughout. In all tumours as well as adjacent non-neoplastic hepatocytes displaying class I

**Figure 1** Histologically normal liver. Intense HLA-A, B, C staining is observed within sinusoids, while hepatocyte membranes show variable faint positivity (arrowed). (Immunoperoxidase × 640).

**Figure 2** Membranous HLA-A, B, C display in hepatocellular carcinoma. Staining is typically uniform throughout the specimen and is more intense than that observed on apparently normal hepatocytes as illustrated in **Figure 1**. (Immunoperoxidase × 323).

**Figure 3** Hepatocellular carcinoma showing diffuse cytoplasmic staining for HLA-A, B, C. The membranous enhancement observed in **Figure 2** is absent. (Immunoperoxidase × 528).

**Figure 4** Hepatocellular carcinoma showing absence of HLA-A, B, C staining. Non-tumorous hepatocytes (arrowed) show an enhanced 'honeycomb' pattern of membranous positivity. (Immunoperoxidase × 400).

**Figure 6** Serial section for comparison with **Figure 5**. Intense and extensive staining of I chain with the Mab VICY1. (Immunoperoxidase × 400).

antigens, staining was considerably more intense than was observed in the control group.

$\beta_2$m staining accompanied HLA-A, -B, -C in all but two cases which were $\beta_2$m negative but showed faint cytoplasmic HLA-A, -B, -C positivity. The pattern of expression was either cytoplasmic or membranous and generally correlated with the heavy chain.

*Class II expression*

Twenty-nine cases (41.4%) were positive for HLA-DR. Staining was invariably heterogeneous (Figure 5). In 10 of these the extent of staining was graded as 1+, 9 were read as 2+, 6 as 3+, and 4 showed 4+ positivity.

All cases positive for HLA-DR also showed VICY1 staining. In addition a further 4 cases were VICY1$^+$/HLA-DR$^-$. In contrast to the relationship between HLA-A, -B, -C and $\beta_2$m, however, I chain staining was invariably far in excess of that observed for its associated MHC product (Figure 6). Thus, of 33 cases (47.1%) staining with VICY1, 4 were graded as 1+, 5 as 2+, 10 as 3+, and 14 showed 4+ positivity.

For both Mabs, staining tended to be heterogenous, with staining intensity varying from cell to cell. In tumours showing a pseudoglandular pattern the HLA-DR positivity was often confined to the apical portion of the cell (Figure 5). Both antigens tended to be intracytoplasmic although in two tumours occasional small foci of membranous HLA-DR expression were noted.

Two cases stained for HLA-DQ in addition to HLA-DR and I chain. Staining was less intense and more focal than was observed for HLA-DR on serial sections.

All tumours showed a mild to moderate T-cell infiltrate, with OKT-4$^+$ cells (helper/inducer T-cells) being slightly in excess of OKT-8$^+$ cells (cytotoxic/suppressor T-cells). T-cells were usually confined to intervening stroma and at the margins of resected lesions. There was little infiltration between tumour cells, and NK cells were extremely scarce in all specimens. No relationship between inflammatory infiltrate and MHC status was demonstrated although in two cases there was enhanced HLA-DR and I chain staining in a periseptal distribution and around clusters of helper/inducer T-cells.

No relationship was observed between expression of class I or class II antigens and tumour differentiation, stage or HBV status (data not shown).

**Figure 5** Hepatocellular carcinoma stained for HLA-DR. Display of the antigen is sparse (arrowed). Note the apical cytoplasmic stain in the pseudoglandular areas, one containing a central bile plug (asterisk). (Immunoperoxidase × 400).

### Discussion

In this study we have examined the patterns of MHC class I and II display in hepatocellular carcinoma. We have also attempted to qualify the relationship between these antigens and their respective invariant chains.

Expression of class I antigens by normal hepatocytes remains controversial. In our series of non-tumorous biopsies there was faint membranous expression of class I antigens by some liver cells. However such expression may represent a facile response of the hepatocyte to a variety of stimuli (Nagafuchi *et al.*, 1985; Fukusato *et al.*, 1986). Biopsies read as histologically normal in this and other studies, and which express HLA-A, -B, -C, may not represent 'normal' liver, with unrecognised immunological factors or agents such as drugs or alcohol promoting the expression of HLA products at the hepatocyte membrane. This may explain the variable findings in the different series reported to date (Saunders *et al.*, 1979; Montano *et al.*, 1982; Ponder *et al.*, 1983; Lautenschlager *et al.*, 1984; Nagafuchi *et al.*, 1985; Fukusato *et al.*, 1986; Nagafuchi & Scheuer, 1986). However, if HLA-A, -B, -C is expressed at all on normal liver cells then it would appear to be at very low levels.

Our results suggest, therefore, that malignant transformation of hepatocytes is characterized by expression of class I antigens that is at least enhanced if not acquired *de novo*, with virtually all tumours in this series showing intense staining for class I antigens. This finding is in keeping with observations reported elsewhere for small series of HCC (Fukusato *et al.*, 1986; Mazzeo *et al.*, 1986).

This contrasts with the situation reported for other human tumours, including colorectal carcinomas in which there is sometimes loss of expression in comparison with the non-neoplastic epithelium (Daar *et al.*, 1982; Csiba *et al.*, 1984; Momburg *et al.*, 1986; van den Ingh *et al.*, 1987). Furthermore it is suggested by some investigators that loss of display is related to progressive de-differentiation and prognosis (Momburg *et al.*, 1986; van den Ingh *et al.*, 1987). This observation has also been made for malignant melanomas in which attenuated or absent class I expression is associated with a high degree of malignancy (Ruiter *et al.*, 1986). We have not observed any relationship between MHC expression and tumour differentiation in HCC.

Class II antigens were expressed in nearly half of the cases in our series. Expression was heterogeneous and often rather sparce, however, and was always associated with VICY1 positivity, although the latter typically showed far more extensive and intense staining than HLA-DR.

The Mab VICY1 is directed at the core protein of the class II-associated I chain proteoglycan (Giacoletto *et al.*, 1986). It is an unusual transmembranous glycoprotein with some structural similarity to certain membrane receptor molecules including the transferrin receptor (Creswell *et al.*, 1987). Its function at this time is unknown although it may participate in one or more of a variety of activities: biosynthesis, transport/recycling, immune mediation or other as yet unrecognised functions (Koch & Harris, 1984; Long, 1985; Giacoletto *et al.*, 1986; Creswell *et al.*, 1987). Although some cases in our series expressed the I chain in the absence of HLA-DR, it may be that this represents tissue selection rather than true absence of HLA-DR, as the class II antigen is expressed extremely sparcely in some cases showing strong VICY1 staining. It is noted, however, that the murine equivalent of the I chain has been demonstrated in the absence of class II antigen in B-lymphoid and myeloid cell lines (Koch & Harris, 1984), and a subset of tumour cells in one case of mediastinal B-cell lymphoma has been reported to be positive for I chain in the absence of HLA-DR and -DQ (Möller *et al.*, 1986). Furthermore, hyperexpression of the I chain has been demonstrated by electrophoretic techniques in Epstein–Barr virus-transformed leukaemic cells (Spiro *et al.*, 1985). To our knowledge the VICY1 antibody has not previously been applied to epithelial neoplasms, and our observations suggest that it may be of value in the study of class II expression in other tumours.

Although HLA-DR expression has been observed in reactive hepatocytes during HBV infection (Fachetti *et al.*, 1985; van den Oord *et al.*, 1986), data for HLA-DQ expression are not available. We believe that the expression of HLA-DQ in some tumours in this series implies that non-neoplastic liver cells may also express this antigen, but in a sequential or dissociated fashion (Sollid *et al.*, 1987).

It has been suggested that the ability to display class II antigens in response to appropriate stimuli may be constitutive to all cells and that it is the failure to express the antigen by a percentage of tumours which represents the 'aberrant' state (Moore & Ghosh, 1987). The observation in two cases that both HLA-DR and I chain staining are enhanced in a periseptal distribution may support this, lymphokines from the adjacent lymphocytic infiltrate being responsible for the enhancement. Conversely, no such accentuation of staining could be demonstrated for the class I antigens and the regular and diffuse nature of antigen expression in all but a minority of cases suggests to us that it represents a constitutive component in malignant transformation of hepatocytes. Whether the lack of display in some tumours represents a pre- or post-translational event is not known.

The MHC antigens function primarily at the cell surface. In a significant minority of malignant hepatocytes expressing HLA-A, -B, -C, and in the vast majority displaying HLA-DR, expression is confined to the cytoplasm. This has also been observed with respect to class I antigens in other tumour systems (Lampert *et al.*, 1985; Kadin, 1980). The effect of this distribution on functional integrity is unknown.

Just as carinogenesis is accepted to be multifactorial in origin, so it is evident that subsequent tumour growth and metastasis is the consequence of numerous complex factors including characteristics of the tumour cell and host-tumour interactions. The host's immune response to the neoplastic cell is one such factor and any modification of the tumour that will enable it to escape immune surveillance could contribute to subsequent growth and metastasis. The possibility that such modification could affect metastatic behaviour has formed the basis of many paradigms involving viral, chemical, or radiation induced cell transformation or neoplasia in culture systems and/or animal models (Goodenow *et al.*, 1985). There is enhancement of metastatic potential in tumours showing loss or attenuation of class I antigens (Schrier *et al.*, 1983), this being reversible by transfection with the appropriate class I gene (Tanaka *et al.*, 1985). Furthermore, modified class I antigens may themselves act as tumour-specific antigens (Philipps *et al.*, 1986), or influence tumour behaviour by interaction with non-MHC genes or by an hormonal effect (Demant, 1986).

MHC expression and tumour behaviour may also be related in some human tumours, including colorectal carcinomas (van den Ingh *et al.*, 1987), melanomas (Ruiter *et al.*, 1986), breast carcinomas (Fleming *et al.*, 1981), small cell carcinoma of lung (Doyle *et al.*, 1985), and neuroblastomas (Whelan *et al.*, 1985). The possibility that this relationship may influence the behaviour of HCC merits some consideration.

HCC is a tumour associated with a uniformly fatal outcome unless detected and resected at an early stage (Kew & Geddes, 1984; Okuda, 1986). Although progress from the time of diagnosis to death is typically rapid (Kew & Geddes, 1984), there is a prolonged subclinical period (Sheu *et al.*, 1985) and the tumour shows an early and sustained proclivity for capsular and blood vessel infiltration (Wakasa *et al.*, 1985; Kew & Paterson, 1985).

Advanced HCCs are typically massive and show extensive vascular infiltration and intrahepatic spread. Despite this, metastases are often confined to clinically insignificant hilar lymph node and pulmonary deposits (Anthony, 1978; Kew & Paterson, 1985), with death resulting from hepatic failure or complications of an associated cirrhosis.

While many other factors may contribute to the metastatic potential of HCC, the possibility that class I expression in the majority of HCCs may restrict extensive extrahepatic tumour spread should be further investigated by the examination of defined intra- and extrahepatic metastases and comparison with the primary lesion.

The Mab VICY1 was a generous gift of Professor W. Knapp, Vienna.

The authors wish to acknowledge the expert technical assistance of Bernadette Smets, Suzanne Taelemans, Erna Van Dessel and Paula Aertsen, and thank Mr M. Rooseleers for preparing the photographs.

The study was supported in part by a grant from the Belgian Fonds voor Geneeskundig Wetenschappelijk Onderzoek.

Dr A. Paterson is supported in part by post-doctoral fellowships from the South African Medical Research Council and the Cecil John Adams Trust.

### References

ANTHONY, P.P. (1979). Hepatic neoplasms. In *Pathology of the Liver,* MacSween *et al.* (eds) p. 387. Churchill Livingstone: Edinburgh.

CRESWELL, P., BLUM, J.S., KELNER, D.N. & MARKS, M.S. (1987). Biosynthesis and processing of class 11 histocompatibility antigens. *CRC Critical Rev. Immunol.,* **7,** 31.

CSIBA, A., WHITWELL, H.L. & MOORE, M. (1984). Distribution of histocompatibility and leucocyte differentiation antigens in normal human colon and in benign and malignant colonic neoplasms. *Br. J. Cancer,* **50,** 699.

DAAR, A.S., FUGGLE, S.V., TING, A. & FABRE, J.W. (1982). Anomalous expression of HLA-DR antigens on human colorectal cancer cells. *J. Immunol.,* **129,** 447.

DAAR, A.S., FUGGLE, S.V., FABRE, J.W., TING, A. & MORRIS, P.J. (1984). The detailed distribution of HLA-A, B, C antigens in normal human organs. *Transplantation,* **38,** 287.

DEMANT, P. (1986). Histocompatibility and the genetics of tumour resistance. *J. Immunogenet.,* **13,** 61.

DOYLE, A., MARTIN, W.J., FUNA, K. & 8 others (1985). Markedly decreased expression of class 1 histocompatibility antigens, protein, and mRNA in human small-cell lung cancer. *J. Exp. Med.,* **161,** 1135.

FACHETTI, F., BONERA, E., ALBERTINI, A., ZORZI, M. & CALLEA, F. (1985). Immunohistochemical patterns of HBcAg, HLA class I and II in different liver diseases with hepatocytic membrane expression of HBsAg. *Hepatology,* **5,** 1049 (*Abstract*).

FESTENSTEIN, H. (1987). The biological consequences of altered MHC expression on tumours. *Br. Med. Bull.,* **43,** 217.

FLEMING, K.A., MCMICHAEL, A., MORTON, J.A., WOODS, J. & MCGEE, J.O.D. (1981). Distribution of HLA class 1 antigens in normal human tissue and in mammary cancer. *J. Clin. Pathol.,* **34,** 779.

FUKUSATO, T., GERBER, M.A., THUNG, S.N., FERRONE, S. & SCHAFFNER, F. (1986). Expression of HLA class 1 antigens on hepatocytes in liver disease. *Am. J. Pathol.,* **123,** 264.

GIACOLETTO, K.S., SANT, A.J., BONO, C. & 4 others (1986). The human invariant chain is the core protein of the human class 11-associated proteoglycan. *J. Exp. Med.,* **164,** 1422.

GOODENOW, R.S., VOGEL, J.M. & LINSK, R.L. (1985). Histocompatibility antigens on murine tumors. *Science,* **230,** 777.

JONES, P.P., MURPHY, D.B., HEWGILL, D. & MCDEVITT, H.O. (1978). Detection of a common polypeptide chain in *I-A* and *I-E* subregion immunoprecipitates. *Immunochemistry,* **16,** 51.

KADIN, M.E. (1980). Ia-like (HLA-DR) antigens in the diagnosis of lymphoma and undifferentiated tumors. *Arch. Pathol. Lab. Med.,* **104,** 503.

KEW, M.C. & GEDDES, E.W. (1982). Hepatocellular carcinoma in rural southern African blacks. *Medicine,* **61,** 98.

KEW, M.C. & PATERSON, A.C. (1985). Unusual clinical presentations of hepatocellular carcinoma. *Trop. Gastroenterol.,* **6,** 10.

KOCH, N. & HARRIS, A.W. (1984). Differential expression of the invariant chain in mouse tumor cells: Relationship to B lymphoid development. *J. Immunol.,* **132,** 12.

LAFUSE, W.P. & DAVID, C.S. (1984). IA antigens: Genes, molecules, and function. *Transplantation,* **38,** 443.

LAMPERT, I.A., KIRKLAND, S., FARRELL, S. & BORYSIEWICZ, L.K. (1985). HLA-DR expression in a human colonic carcinoma cell line. *J. Pathol.,* **146,** 337.

LAUTENSCHLAGER, I., TASKINEN, E., INKINEN, K., LEHTO, V.-P., VIRTANEN, I. & HAYRY, P. (1984). Distribution of the major histocompatibility complex antigens on different cellular components of human liver. *Cell Immunol.,* **85,** 191.

LONG, E.O. (1985). In search of a function for the invariant chain associated with Ia antigens. *Surv. Immunol. Res.,* **4,** 27.

MAZZEO, V., MIGLIO, F., BARALDINI, M. & 7 others (1986). Aberrant expression of HLA class I and II antigens in hepatocellular carcinoma. *Ital. J. Gastroenterol.,* **18,** 67 (*Abstract*).

MÖLLER, P., LÄMMLER, B., HERRMANN, B., OTTO, H.F., MOLDENHAUER, G. & MOMBURG, F. (1986). The primary mediastinal clear cell lymphoma of B-cell type has variable defects in MHC antigen expression. *Immunology,* **59,** 411.

MOMBURG, F., DEGENER, T., BACCHUS, E., MOLDENHAUER, G., HÄMMERLING, G.J. & MÖLLER, P. (1986). Loss of HLA-A, B, C and *de novo* expression of HLA-D in colorectal cancer. *Int. J. Cancer,* **37,** 179.

MONTANO, L., MIESCHER, G.C., GOODALL, A.H., WIEDMANN, K.H., JANOSSY, G. & THOMAS, H.C. (1982). Hepatitis B virus and HLA antigen display in the liver during chronic hepatitis B virus infection. *Hepatology,* **2,** 557.

MOORE, M. & GHOSH, A.K. (1987). 'Aberrant' MHC class II expression in epithelia. *Lancet,* **i,** 165.

NAGAFUCHI, Y., HOBBS, K.E.F., THOMAS, H.C. & SCHEUER, P.J. (1985). Expression of beta-2-microglobulin on hepatocytes after liver transplantation. *Lancet,* **i,** 551.

NAGAFUCHI, Y. & SCHEUER, P.J. (1986). Expression of $\beta_2$-microglobulin on hepatocytes in acute and chronic type B hepatitis. *Hepatology,* **6,** 20.

NATALI, P.G., DE MARTINO, C., QUARANTA, V., BIGOTTI, A., PELLEGRINO, M.A. & FERRONE, S. (1981). Changes in Ia-like antigen expression on malignant human cells. *Immunogenetics,* **12,** 409.

OKUDA, K. (1986). Early recognition of hepatocellular carcinoma. *Hepatology,* **6,** 729.

PHILIPPS, C., MCMILLAN, M., FLOOD, P.M. & 6 others (1985). Identification of a unique tumor-specific antigen as a novel class 1 major histocompatibility molecule. *Proc. Natl Acad. Sci. USA,* **82,** 5140.

PONDER, B.A.J., WILKINSON, M.M., WOOD, M. & WESTWOOD, J.H. (1983). Immunohistochemical demonstration of H2 antigens in mouse tissue sections. *J. Histochem. Cytochem.,* **31,** 911.

QUARANTA, V., MAJDIC, O., STINGL, G., LISZKA, K., HONIGSMANN, H. & KNAPP, W. (1984). A human Ia cytoplasmic determinant located on multiple forms of invariant chain ($\gamma^1$, $\gamma^2$, $\gamma^3$). *J. Immunol.,* **132,** 1900.

RUITER, D., BROCKER, E.-B. & FERRONE, S. (1986). Expression and susceptibility to modulation by interferons of HLA class I and II antigens on melanoma cells: Immunochemical analysis and clinical relevance. *J. Immunogenet.,* **13,** 229.

SAUNDERS, D.A., BEALS, T.F. & SCHULTZ, J.S. (1979). Qualitative and quantitative evaluation of indirect immunofluorescent H-2 stain on tissue sections. *Tissue Antigens,* **14,** 73.

SCHRIER, P.I., BERNARDS, R., VAESSEN, R.T.M.J., HOUWELING, A. & VAN DER EB, A.J. (1983). Expression of class I major histocompatibility antigens switched off by highly oncogenic adenovirus 12 in transformed rat cells. *Nature,* **305,** 771.

SHEU, J.-C., SUNG, J.-L., CHEN, D.-S. & 9 others (1985). Growth rate of asymptomatic hepatocellular carcinoma and its clinical implications. *Gastroenterology,* **89,** 259.

SOLLID, L.M., GAUDERNACK, G., MARKUSSEN, G., KVALE, D., BRANDTZAEG, P. & THORSBY, E. (1987). Induction of various HLA class II molecules in a human colonic adenocarcinoma cell line. *Scand. J. Immunol.,* **25,** 175.

SPIRO, R.C., SAIRENJI, T. & HUMPHREYS, R.E. (1985). Kinetics of Ii synthesis, processing and turnover in *n*-butyrate-treated Burkitt's lymphoma cell lines which express or do not express class II antigens and in hairy leukemic cells. *J. Immunol.,* **134,** 3539.

STRACHAN, T. (1987). Molecular genetics and polymorphism of class I HLA antigens. *Br. Med. Bull.,* **43,** 1.

TANAKA, K., ISSELBACHER, K.J., KHOURY, G. & JAY, G. (1985). Reversal of oncogenesis by the expression of a major histocompatibility complex class I gene. *Science,* **228,** 26.

VAN DEN INGH, H.F., RUITER, D.J., GRIFFIOEN, G., VAN MUIJEN, G.N.P. & FERRONE, S. (1987). HLA antigens in colorectal tumours – low expression of HLA class I antigens in mucinous colorectal carcinomas. *Br. J. Cancer,* **55,** 125.

VAN DEN OORD, J.J., DE VOS, R. & DESMET, V.J. (1986). *In situ* distribution of major histocompatibility complex products and viral antigens in chronic hepatitis B virus infection: Evidence that HBc-containing hepatocytes may express HLA-DR antigens. *Hepatology,* **6,** 981.

WAKASA, K., SAKURAI, M., OKAMURA, J. & KURODA, C. (1985). Pathological study of small hepatocellular carcinoma: Frequency of their invasion. *Virchows Arch* [*Pathol. Anatl*]., **407,** 259.

WHELAN, J.P., CHATTEN, J. & LAMPSON, L.A. (1985). HLA class I and $\beta_2$-microglobulin expression in frozen and formaldehyde-fixed paraffin sections of neuroblastoma tumors. *Cancer Res.,* **45,** 5976.

WORLD HEALTH ORGANIZATION (1978). *Tumours of the Liver.* Geneva: Switzerland.


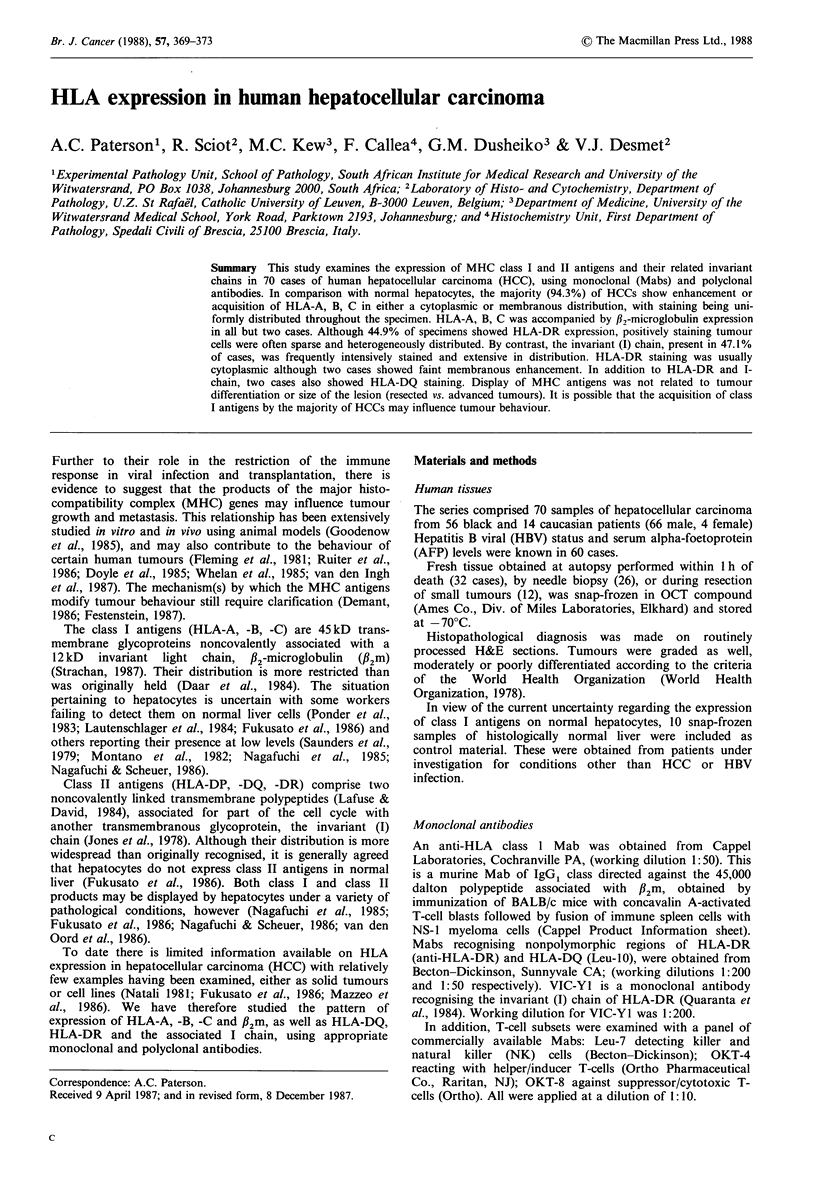

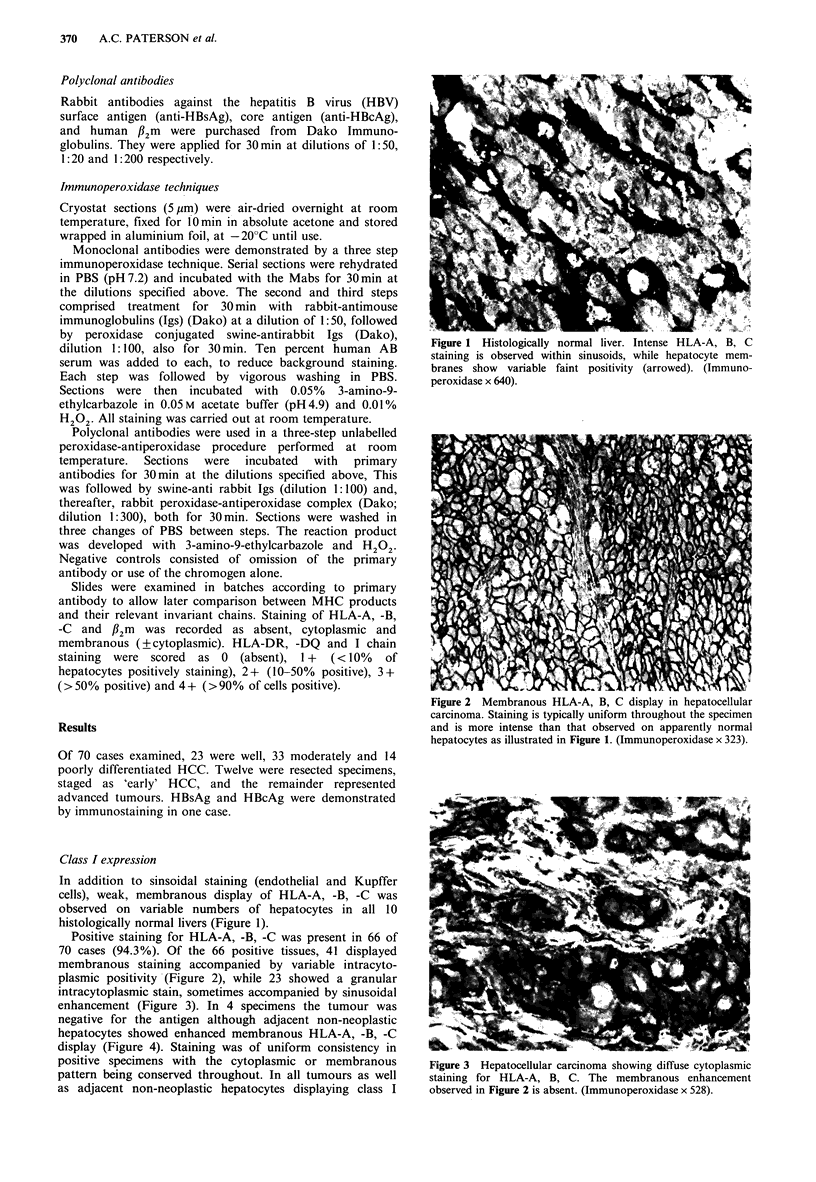

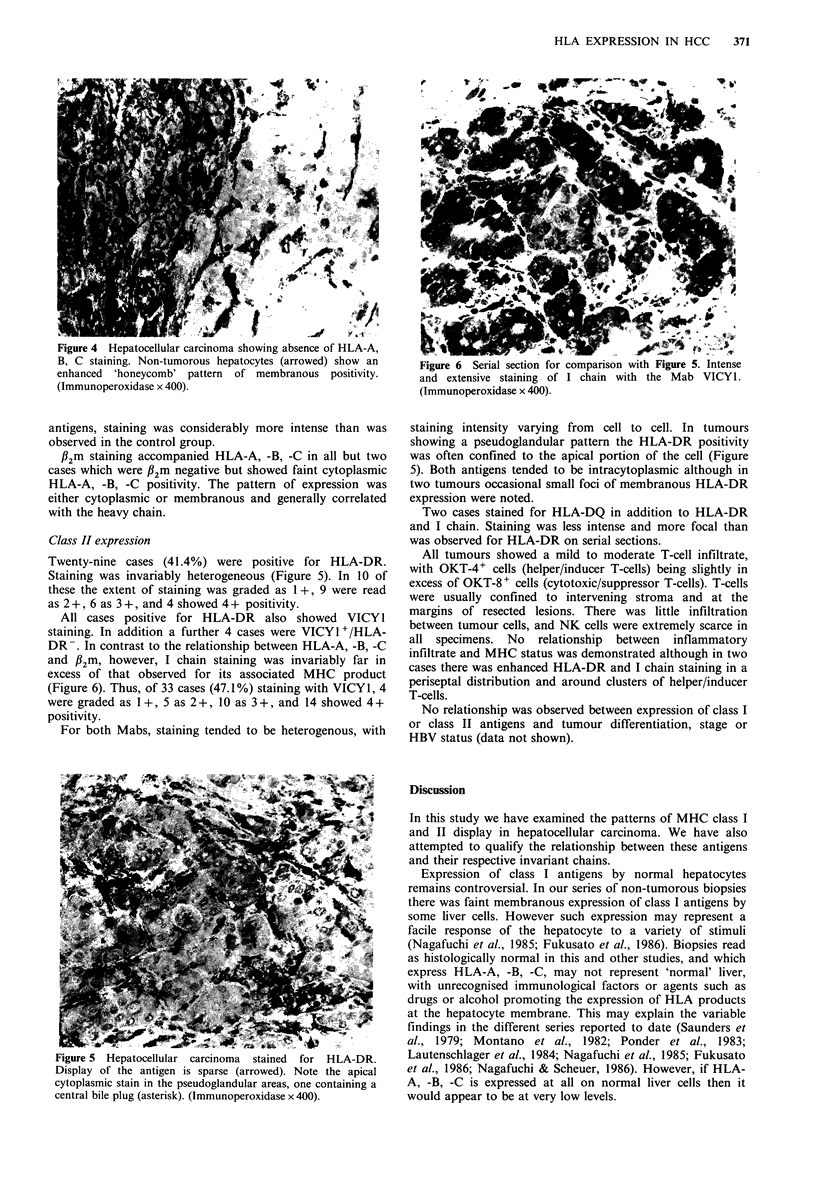

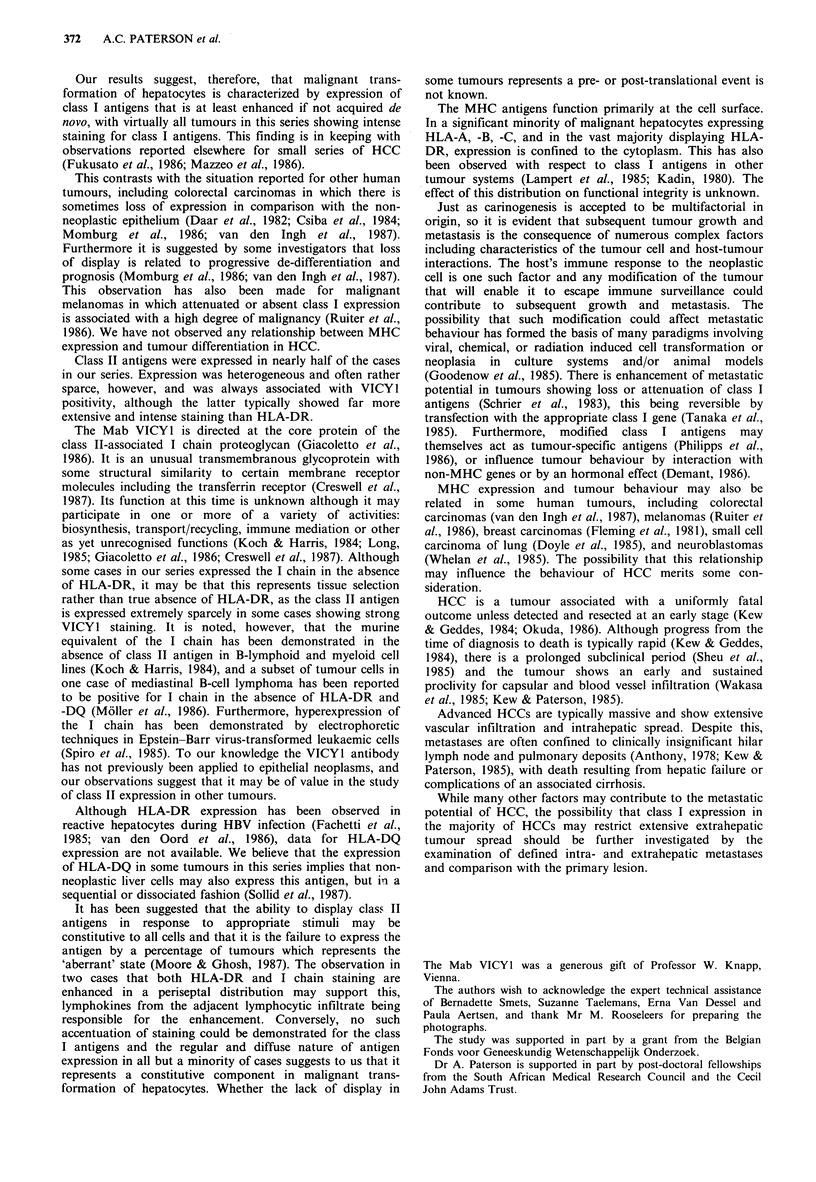

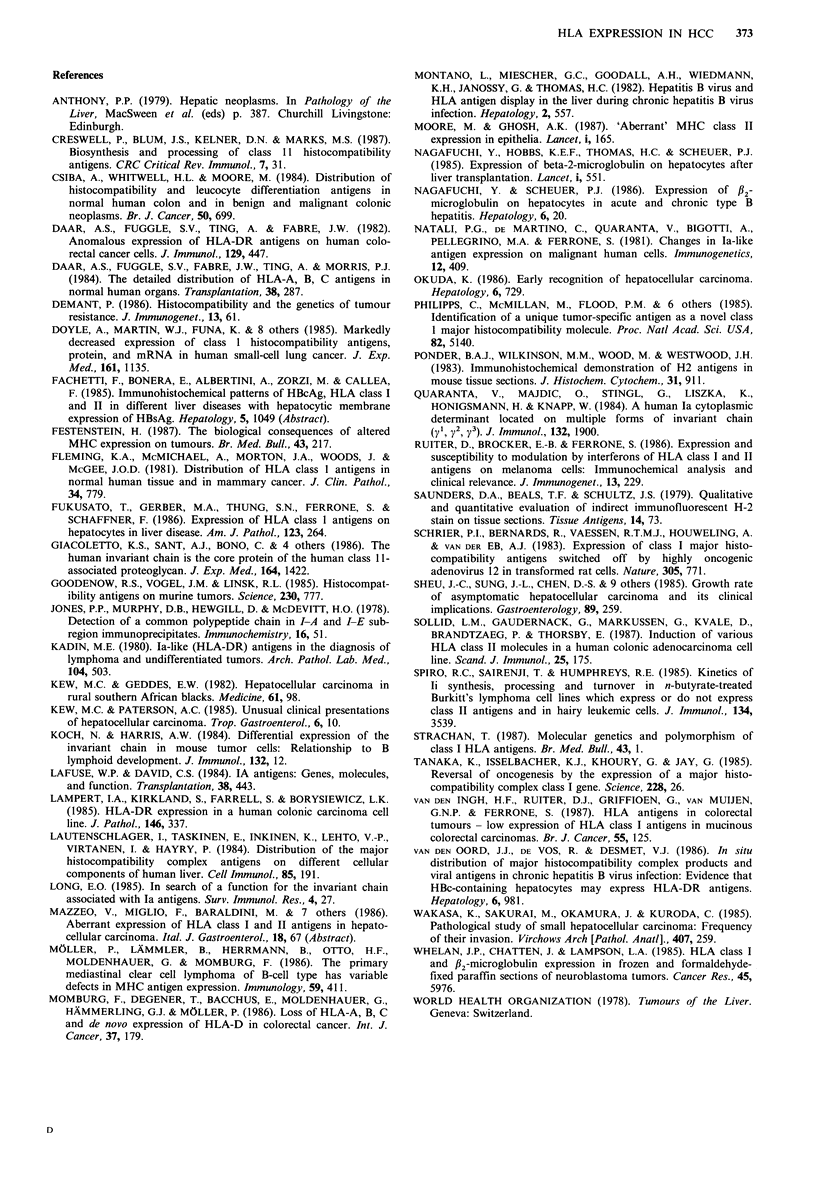


## References

[OCR_00505] Cresswell P., Blum J. S., Kelner D. N., Marks M. S. (1987). Biosynthesis and processing of class II histocompatibility antigens.. Crit Rev Immunol.

[OCR_00510] Csiba A., Whitwell H. L., Moore M. (1984). Distribution of histocompatibility and leucocyte differentiation antigens in normal human colon and in benign and malignant colonic neoplasms.. Br J Cancer.

[OCR_00521] Daar A. S., Fuggle S. V., Fabre J. W., Ting A., Morris P. J. (1984). The detailed distribution of HLA-A, B, C antigens in normal human organs.. Transplantation.

[OCR_00516] Daar A. S., Fuggle S. V., Ting A., Fabre J. W. (1982). Anomolous expression of HLA-DR antigens on human colorectal cancer cells.. J Immunol.

[OCR_00526] Demant P. (1986). Histocompatibility and the genetics of tumour resistance.. J Immunogenet.

[OCR_00530] Doyle A., Martin W. J., Funa K., Gazdar A., Carney D., Martin S. E., Linnoila I., Cuttitta F., Mulshine J., Bunn P. (1985). Markedly decreased expression of class I histocompatibility antigens, protein, and mRNA in human small-cell lung cancer.. J Exp Med.

[OCR_00542] Festenstein H. (1987). The biological consequences of altered MHC expression on tumours.. Br Med Bull.

[OCR_00546] Fleming K. A., McMichael A., Morton J. A., Woods J., McGee J. O. (1981). Distribution of HLA class 1 antigens in normal human tissue and in mammary cancer.. J Clin Pathol.

[OCR_00552] Fukusato T., Gerber M. A., Thung S. N., Ferrone S., Schaffner F. (1986). Expression of HLA class I antigens on hepatocytes in liver disease.. Am J Pathol.

[OCR_00557] Giacoletto K. S., Sant A. J., Bono C., Gorka J., O'Sullivan D. M., Quaranta V., Schwartz B. D. (1986). The human invariant chain is the core protein of the human class II-associated proteoglycan.. J Exp Med.

[OCR_00562] Goodenow R. S., Vogel J. M., Linsk R. L. (1985). Histocompatibility antigens on murine tumors.. Science.

[OCR_00566] Jones P. P., Murphy D. B., Hewgill D., McDevitt H. O. (1979). Detection of a common polypeptide chain in I--A and I--E sub-region immunoprecipitates.. Mol Immunol.

[OCR_00571] Kadin M. E. (1980). Ia-like (HLA-DR) antigens in the diagnosis of lymphoma and undifferentiated tumors.. Arch Pathol Lab Med.

[OCR_00576] Kew M. C., Geddes E. W. (1982). Hepatocellular carcinoma in rural southern African blacks.. Medicine (Baltimore).

[OCR_00580] Kew M. C., Paterson A. C. (1985). Unusual clinical presentations of hepatocellular carcinoma.. Trop Gastroenterol.

[OCR_00584] Koch N., Harris A. W. (1984). Differential expression of the invariant chain in mouse tumor cells: relationship to B lymphoid development.. J Immunol.

[OCR_00589] Lafuse W. P., David C. S. (1984). Ia antigens: genes, molecules and function.. Transplantation.

[OCR_00593] Lampert I. A., Kirkland S., Farrell S., Borysiewicz L. K. (1985). HLA-DR expression in a human colonic carcinoma cell line.. J Pathol.

[OCR_00598] Lautenschlager I., Taskinen E., Inkinen K., Lehto V. P., Virtanen I., Häyry P. (1984). Distribution of the major histocompatibility complex antigens on different cellular components of human liver.. Cell Immunol.

[OCR_00604] Long E. O. (1985). In search of a function for the invariant chain associated with Ia antigens.. Surv Immunol Res.

[OCR_00619] Momburg F., Degener T., Bacchus E., Moldenhauer G., Hämmerling G. J., Möller P. (1986). Loss of HLA-A,B,C and de novo expression of HLA-D in colorectal cancer.. Int J Cancer.

[OCR_00625] Montano L., Miescher G. C., Goodall A. H., Wiedmann K. H., Janossy G., Thomas H. C. (1982). Hepatitis B virus and HLA antigen display in the liver during chronic hepatitis B virus infection.. Hepatology.

[OCR_00631] Moore M., Ghosh A. K. (1987). "Aberrant" MHC class II expression in epithelia.. Lancet.

[OCR_00613] Möller P., Lämmler B., Herrmann B., Otto H. F., Moldenhauer G., Momburg F. (1986). The primary mediastinal clear cell lymphoma of B-cell type has variable defects in MHC antigen expression.. Immunology.

[OCR_00635] Nagafuchi Y., Hobbs K. E., Thomas H. C., Scheuer P. J. (1985). Expression of beta-2-microglobulin on hepatocytes after liver transplantation.. Lancet.

[OCR_00640] Nagafuchi Y., Scheuer P. J. (1986). Expression of beta 2-microglobulin on hepatocytes in acute and chronic type B hepatitis.. Hepatology.

[OCR_00645] Natali P. G., De Martino C., Quaranta V., Bigotti A., Pellegrino M. A., Ferrone S. (1981). Changes in Ia-like antigen expression on malignant human cells.. Immunogenetics.

[OCR_00651] Okuda K. (1986). Early recognition of hepatocellular carcinoma.. Hepatology.

[OCR_00655] Philipps C., McMillan M., Flood P. M., Murphy D. B., Forman J., Lancki D., Womack J. E., Goodenow R. S., Schreiber H. (1985). Identification of a unique tumor-specific antigen as a novel class I major histocompatibility molecule.. Proc Natl Acad Sci U S A.

[OCR_00661] Ponder B. A., Wilkinson M. M., Wood M., Westwood J. H. (1983). Immunohistochemical demonstration of H2 antigens in mouse tissue sections.. J Histochem Cytochem.

[OCR_00666] Quaranta V., Majdic O., Stingl G., Liszka K., Honigsmann H., Knapp W. (1984). A human Ia cytoplasmic determinant located on multiple forms of invariant chain (gamma, gamma 2, gamma 3).. J Immunol.

[OCR_00672] Ruiter D. J., Bröcker E. B., Ferrone S. (1986). Expression and susceptibility to modulation by interferons of HLA class I and II antigens on melanoma cells. Immunohistochemical analysis and clinical relevance.. J Immunogenet.

[OCR_00678] Saunders D. A., Beals T. F., Schultz J. S. (1979). Qualitative and quantitative evaluation of indirect immunofluorescent H-2 stain on tissue sections.. Tissue Antigens.

[OCR_00683] Schrier P. I., Bernards R., Vaessen R. T., Houweling A., van der Eb A. J. Expression of class I major histocompatibility antigens switched off by highly oncogenic adenovirus 12 in transformed rat cells.. Nature.

[OCR_00689] Sheu J. C., Sung J. L., Chen D. S., Yang P. M., Lai M. Y., Lee C. S., Hsu H. C., Chuang C. N., Yang P. C., Wang T. H. (1985). Growth rate of asymptomatic hepatocellular carcinoma and its clinical implications.. Gastroenterology.

[OCR_00694] Sollid L. M., Gaudernack G., Markussen G., Kvale D., Brandtzaeg P., Thorsby E. (1987). Induction of various HLA class II molecules in a human colonic adenocarcinoma cell line.. Scand J Immunol.

[OCR_00700] Spiro R. C., Sairenji T., Humphreys R. E. (1985). Kinetics of Ii synthesis, processing, and turnover in n-butyrate-treated Burkitt's lymphoma cell lines which express or do not express class II antigens and in hairy leukemic cells.. J Immunol.

[OCR_00707] Strachan T. (1987). Molecular genetics and polymorphism of class I HLA antigens.. Br Med Bull.

[OCR_00711] Tanaka K., Isselbacher K. J., Khoury G., Jay G. (1985). Reversal of oncogenesis by the expression of a major histocompatibility complex class I gene.. Science.

[OCR_00729] Wakasa K., Sakurai M., Okamura J., Kuroda C. (1985). Pathological study of small hepatocellular carcinoma: frequency of their invasion.. Virchows Arch A Pathol Anat Histopathol.

[OCR_00734] Whelan J. P., Chatten J., Lampson L. A. (1985). HLA class I and beta 2-microglobulin expression in frozen and formaldehyde-fixed paraffin sections of neuroblastoma tumors.. Cancer Res.

[OCR_00716] van den Ingh H. F., Ruiter D. J., Griffioen G., van Muijen G. N., Ferrone S. (1987). HLA antigens in colorectal tumours--low expression of HLA class I antigens in mucinous colorectal carcinomas.. Br J Cancer.

[OCR_00722] van den Oord J. J., de Vos R., Desmet V. J. (1986). In situ distribution of major histocompatibility complex products and viral antigens in chronic hepatitis B virus infection: evidence that HBc-containing hepatocytes may express HLA-DR antigens.. Hepatology.

